# Metformin induces ferroptosis through the Nrf2/HO-1 signaling in lung cancer

**DOI:** 10.1186/s12890-023-02655-6

**Published:** 2023-09-25

**Authors:** Chengmin Deng, Lin Xiong, Yang Chen, Kaifeng Wu, Jie Wu

**Affiliations:** 1 Scientific Research Center, The First People’s Hospital of Zunyi (The Third Affiliated Hospital of Zunyi Medical University), Zunyi, Guizhou China; 2 Department of Clinical Laboratory, The First People’s Hospital of Zunyi (The Third Affiliated Hospital of Zunyi Medical University), Zunyi, Guizhou China; 3 College of Basic Medicine, Zunyi Medical University, Zunyi, Guizhou China

**Keywords:** Metformin, Ferroptosis, Lung cancer, Oxidative stress, Nrf2/HO-1 signaling

## Abstract

**Background:**

Metformin is the most frequently prescribed medication for the treatment of type II diabetes mellitus and has played an anti-tumor potential in a variety of cancer types. Metformin can inhibit the growth of many cancer cells through various mechanisms, including ferroptosis. However, it is still unclear whether metformin can induce ferroptosis in lung cancer.

**Methods:**

This study evaluated the anti-tumor effect of metformin by detecting the levels of oxidative stress factors, the levels of ferrous ions, and the expression of ferroptosis-related genes in A549 and H1299 lung cancer cell lines treated with or without metformin.

**Results:**

The results showed that metformin treatment increased the levels of MDA, ROS and iron ions, while decreased the levels of GSH, T-SOD and CAT. Meanwhile, metformin treatment reduced the protein expression levels of Gpx4 and SLC7A11, Nrf2 and HO-1, while the addition of ferroptosis inhibitor ferrostatin-1 reversed the reduction.

**Conclusions:**

These results demonstrated that metformin exerts anti-tumor effects by inducing ferroptosis through the Nrf2/HO-1 signaling pathway in lung cancer cells, providing a theoretical basis for drug therapy of lung cancer patients.

**Supplementary Information:**

The online version contains supplementary material available at 10.1186/s12890-023-02655-6.

## Background

The incidence of lung cancer is continually rising worldwide. In accordance with estimates of the International Agency for Research on Cancer (IARC), lung cancer ranks second in morbidity and first in mortality [1]. Since the symptoms of lung cancer are not obvious in the early stages, most patients are diagnosed in the middle and late stages, the 5-year survival rate after surgery has always hovered between20% and 30% [2]. Treatment options for lung cancer include surgery, radiotherapy, chemotherapy, targeted therapies and combination of the different treatment methods [3]. Although these treatments have significantly improved the prognosis of most lung cancer patients, the prognosis is still unsatisfactory, especially in advanced-stage patients. With the intensification of industrialization, environmental pollution has become increasingly serious, and the morbidity and mortality of lung cancer have increased in recent years, which causing serious medical and social burden [4]. Therefore, the research on the treatment of lung cancer is still intensively demanded.

Metformin, a first-line drug for treating type 2 diabetes mellitus, was reported to improve the symptoms of inflammation, inflammatory bowel disease, obesity, osteoporosis, periodontitis, polycystic ovarian syndrome, cancer, neurodegeneration, etc. [5]. Metformin may reduce the risk of cancer in patients with type 2 diabetes and inhibit the cell growth of various cancers, including pancreatic cancer, colon cancer, prostate cancer, ovarian cancer and breast cancer [6]. Clinical investigation studies have shown that the use of metformin was associated with a reduced risk and a good prognosis of lung cancer [7–9]. Metformin inhibits the proliferation, colony formation, and induces apoptosis in lung cancer cells [10]. In vivo studies show that metformin treatment can inhibit the activation of various tyrosine kinase receptors (EGFR, IGFR and VEGFR) and the phosphorylation of mTOR through AMPK-dependent and independent mechanisms, thus inhibiting the occurrence and metastasis of lung cancer [10, 11]. Metformin has also been shown to inhibit epithelial-mesenchymal transformation (EMT) and inhibit the proliferation of tumor cells [12]. It is noteworthy that metformin could amplify cell death mechanisms, especially apoptosis in a broad-spectrum of cancer cells [13]. However, emerging evidence has illustrated that several cancer cell lines are chemoresistant due to defects in apoptotic cell death. Hence, treatment strategies that bypass apoptotic cell death mechanism and emphasize the non-apoptotic cell death mechanism, such as pyroptosis, necroptosis, and ferroptosis, seems to exhibit effective therapeutic potentials to mitigate chemoresistance [14].

Ferroptosis is a newly identified iron-dependent and reactive oxygen species- (ROS-) dependent form of non-necrosic, non-autophagic and non-apoptotic programmed cell death in various pathophysiological states, including cancer and that targeting ferroptosis has been considered as a novel anti-cancer strategy [15–17]. A variety of anticancer drugs can inhibit ferroptosis-related molecules and channels to induce ferroptotic cancer cell death, such as GPX4 and System Xc-, and then inhibit the growth of tumor cells [18]. There are more and more studies on the treatment of cancer by inducing ferroptosis, including breast cancer, pancreatic cancer, lung cancer, ovarian cancer, brain tumor, hepatocellular carcinoma and fibrosarcoma [19]. Studies have shown that ferroptosis inducers Erastin and RSL3 can regulate cellular sensitivity to ferroptosis, inhibiting the growth and metastasis of lung cancer cells by inhibiting the expression of System Xc- and GPX [20]. Metformin induces ferroptosis by targeting the miR-324-3p/GPX4 axis or by inhibiting autophagy via lncRNA H19 in breast cancer [21, 22]. In liver cancer cells, combination of metformin and sorafenib induces ferroptosis via p62-Keap1-Nrf2 Pathway [23].

Based on the above elaboration, metformin may be potential anticancer agent by inducing ferroptotic cancer cell death. However, to the best of our knowledge, there is still no report on whether metformin can induce ferroptosis in lung cancer cells. In this study, the protein expression levels of the Nrf2/HO-1 redox signaling pathway and the oxidative/antioxidative ferroptosis-relative factors were evaluated to explore the metformin induced ferroptosis mechanism in lung cancer cells, providing a new strategy for the treatment of lung cancer.

## Methods

### Cell lines and culture condition

A549 and H1299 cells (Cell Bank of the Typical Culture Preservation Committee, Chinese Academy of Sciences) were cultured in RPMI-1640 medium (Gibco, China ) supplemented with 10% fetal bovine serum (FBS) (Biological Industries, Israel) and 100 µg/mL PenStrep. The cells cultured at 37 °C in 5% CO_2_ humidified chamber were passaged when they grew to 80% confluence. Cells were inoculated into six-well plates with 1 × 10^6^ cells per well and cultured under normal conditions for 48 h. Subsequently, the cells were treated with 0.5 mM, 1.0 mM or 2.0 mM metformin [24] (Met, S1950, Selleck, China) for 48 h with or without the supplement of 10 µM Ferrostatin-1 (Fer-1, S7243, Selleck, China).

### Cell viability assay

Cell viability was assessed via CCK-8 assay (CK04, Dojindo Laboratories, Japan). Briefly, about 5 × 10^3^ cells were added into 96-well plates, cultured for 48 h, and the cells were cultured in new medium in the presence of 0.5 mM, 1.0 mM or 2.0 mM metformin for 48 h. Subsequently, 10 µL per well of CCK-8 reagent was added into the culture medium and cells were incubated for an additional 1 h. The absorbance of each well was measured at 450 nm using a BioTek microplate reader. Wells containing culture medium and the CCK-8 reagents without cells were used as the blank control. All the tests were repeated at least three times.

### Reactive oxygen species (ROS), Malondialdehyde (MDA), reduced glutathione (GSH) and Total Superoxide Dismutase (T-SOD), Catalase (CAT) assay

ROS production was measured using an ROS assay kit (E004-1-1, Nanjing Jiangcheng, China). After the cells in the 6-well plates were treated with different concentrations of metformin, the cells were washed with PBS for three times. Subsequently, 1mL of serum free medium containing 10 µM of 2’-7’-dichlorodihydrofluorescein diacetate (DCFH-DA) probe was added into each well and the cells were incubated at 37 °C for 30 min. The six-well plates were imaged by fluorescence microscopy. Then the cells resuspended with 1mL PBS were transferred to 1.5mL EP tube, and were resuspended and washed twice with PBS, and finally resuspended with 200 µL PBS. The cell suspensions were transferred to a 96-well black-walled plate. Fluorescence was measured at an excitation wavelength of 488 nm and an emission wavelength of 520 nm.

The contents of MDA, GSH, and T-SOD, CAT in cells treated with different concentration of metformin were measured using MDA assay kit (A003-4-1, Nanjing Jiangcheng, China), GSH assay kit (A003-2-1, Nanjing Jiangcheng, China), T-SOD activity assay kit (E-BC-K020-M, Elabscience, China), CAT activity assay kit (E-BC-K031-M, Elabscience, China) according to the manufacturer’s instructions, respectively. All experiments were repeated at least three times.

### Fe^2+^ iron amount assay

The level of ferrous iron was tested by a ferrous iron colorimetric assay kit (E-BC-K773-M, Elabscience, China). Briefly, after washed with serum-free medium for three times, and treated with a final concentration of 1 µM FerroOrange (F374, Dojindo Laboratories, Japan); the cells were cultured in the incubator for additional 30 min and directly examined by fluorescence microscope. According to the manufacturer’s instructions, proper amount of cells was added to iron assay buffer I, homogenized on ice, and centrifuged at 12,000 × g for 10 min at 4 °C to obtain the supernatant for the assay. A volume of 80 µL supernatant (or 80 µL standards with different concentrations for making standard curves) was incubated with 80 µL of assay buffer II in a 96-well microplate at 37 °C in the dark for 10 min. The absorbance was measured at 593 nm with a Biotech microplate reader.

### RT-qPCR analysis

Total RNA was prepared by RNAiso Plus (9109, Takara, Japan) and reverse transcribed to cDNA by PrimeScript™ RT reagent Kit (RR037A, Takara, Japan). ArtiCanCEO SYBR RT-qPCR Mix (TSE401, Tsingke, China) was used to analyze the mRNA expression. The sequences of all RT-qPCR primers used in our research are listed in Table 1. The 2^−ΔΔCt^ method was used to analyze the RT-qPCR results.

**Table 1:** Sequences of primers used for RT-qPCR analysis

Gene	Forward (5′–3′)	Reverse (5′–3′)
GAPDH	CTCCAAAATCAAGTGGGGCG	ATGGTTCACACCCATGACGA
Gpx4	CCGCTGTGGAAGTGGATGAAGATC	GCAGCCGTTCTTGTCGATGAGG
SLC7A11	TCTCCAAAGGAGGTTACCTGC	AGACTCCCCTCAGTAAAGTGAC
Nrf2	GTGTGGCATCACCAGAACAC	GACACTTCCAGGGGCACTAT
HO-1	AAGACTGCGTTCCTGCTCAAC	AAAGCCCTACAGCAACTGTCG

### Western-blot assay

Western blot was performed as previously described [25]. Briefly, after washed twice with PBS, 200 µL ice cold RIPA buffer (R0010, Solarbio, China) with PMSF (P0100; Solarbio, China) and Protein Phosphatase Inhibitor (P1260, Solarbio, China) was added to each well, the cells were scraped and transferred into a microcentrifuge tube and lysed on ice for 30 min. Cell lysates were centrifuged at 12,000 × g at 4 °C for 30 min and the supernatant was collected for analysis. Protein concentrations were measured by BCA protein assay kit (PC0020, Solarbio, China). Total 20 µg protein per sample was mixed with 5 × loading buffer, denatured at 95 °C for 10 min, separated on 10% SDS-PAGE and then transferred onto polyvinylidene difluoride (PVDF) membranes (IPVH00010, Millipore, USA). The membranes were blocked with 5% skimmed milk in TBST buffer (137 mM NaCl, 25 mM Tris-HCl, 0.05% Tween-20, pH 7.5) for 1 h at room temperature and followed by incubation with specific primary antibodies overnight at 4 °C (anti-Gpx4, cat: 67763-1-lg, dilution: 1:1000, Proteintech, China; anti-SLC7A11, cat: Ab300667, dilution: 1:2000, Abcam, USA; anti-HO-1, cat: 10701-1-AP, dilution: 1:2000, Proteintech, China; anti-Nrf2, cat: 66504-1-lg, dilution: 1:2000, Proteintech, China; anti-β-actin, cat: 20536-1-AP, dilution: 1:6000, Proteintech, China). Membranes were washed three times with TBST to remove the primary antibodies, then incubated with HRP-conjugated secondary antibodies goat anti-rabbit IgG (H + L) (cat: SA00001-2; 1:5000 and goat anti-mouse IgG (H + L) (cat: SA00001-1, 1:5000; both from Proteintech, China) on a shaking bed for 2 h at room temperature. After washing 3 times with TBST, the signals were visualized via Omni-ECL™ Femto Light Chemiluminescence Kit (SQ201, Epizyme Biomedical, China), and acquired with a ChemiDoc™ XRS system (Bio-Rad). The images were analyzed by ImageJ 1.45 software (NIH) and β-actin was used as the internal control. Each assay was repeated 4 times.

### Statistical analysis

All data are presented as the means ± standard deviations (SDs) of at least three separate experiments. The Shapiro-Wilk test was applied to ascertain whether the data were normally distributed. Two groups of normally distributed data were compared using SPSS 19.0, and a one-way ANOVA followed by LDS test was performed to investigate any significant group-to-group differences. Nonparametric tests were used for data that were not normally distributed. Statistical significance was defined as a *P*-value of less than 0.05.

## Results

### Metformin reduces the survival of lung cancer cells

**Fig. 1:**
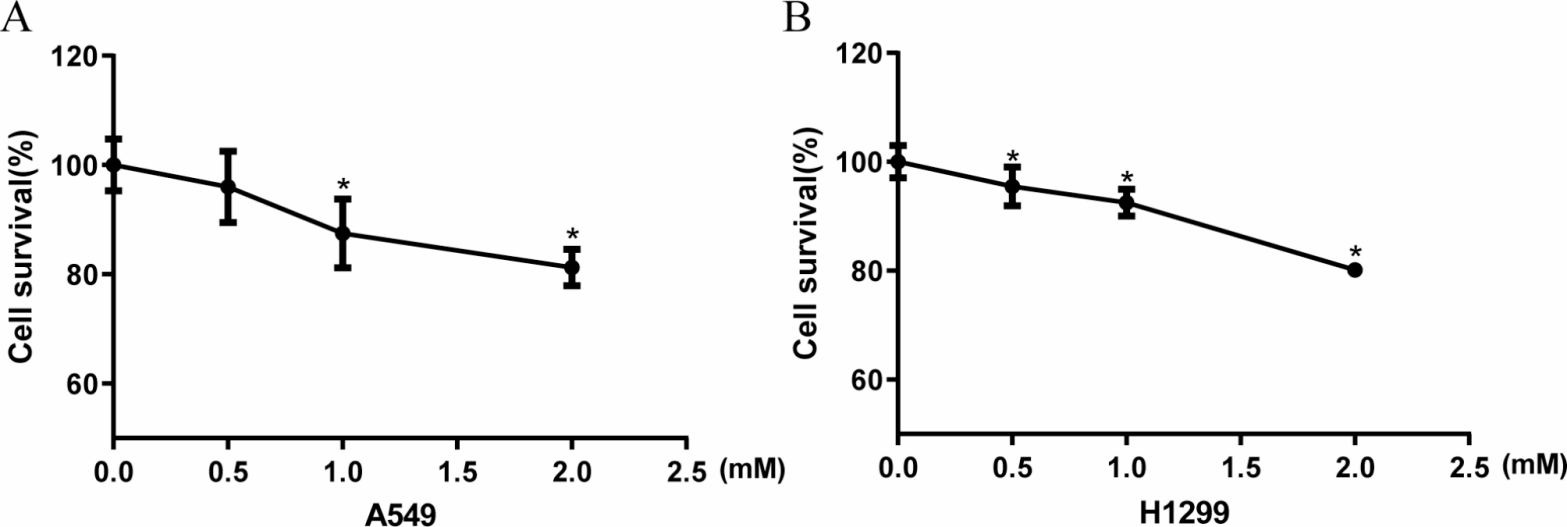
Effects of metformin on survival of A549 and H1299 lung cancer cells. The cell survival rate was detected by CCK-8 assay after 48 h of treatment with different concentrations of metformin in A549 cells **(A)** or H1299 cells **(B)**, n = 3, ^*^P < 0.05 compared with the control group

We first determined the effect of metformin on the viability of lung cancer cells. To illustrate that the effect of metformin is not specific to a single cell line, we used two different lung cancer cells. Results showed that exposure of A549 cells to 1mM of metformin for 48 h could significantly reduce the survival of cells, and with the increase of metformin concentration, the survival rate of A549 cells decreased gradually (Fig. 1A). Similarly, treatment of H1299 cells with 0.5mM metformin for 48 h significantly reduced the survival rate of H1299 cells, and with the increase of metformin concentration, the cell survival rate decreased gradually (Fig. 1B). These results indicate that Met treatment reduces the survival of lung cancer cells in concentration-dependent manner.

### Metformin induces oxidative stress in lung cancer cells

**Fig. 2:**
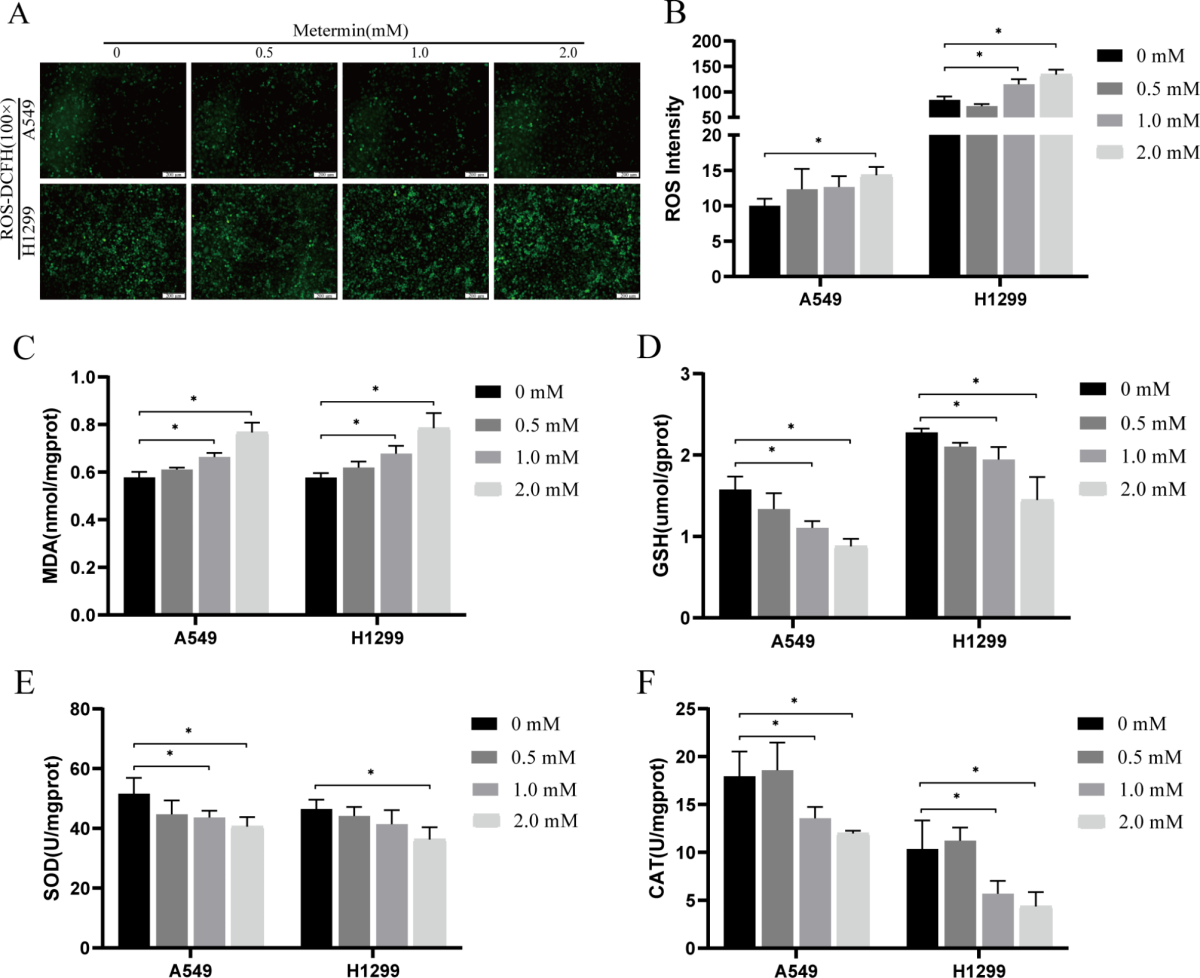
Effects of metformin on the oxidative stress in lung cancer cells. Cells were treated with different concentrations of metformin for 48 h, the intracellular ROS intensity **(A, B)**, the MDA concentration **(C)**, GSH concentration **(D)** and T-SOD concentration **(E)**, CAT concentration **(F)** were determined. n = 3, ^*^P < 0.05 compared with the control group

To determine the level of oxidative stress induced by metformin, the levels of ROS, MDA, GSH and the contents of T -SOD, CAT were determined. Compared with the control group, the levels of intracellular ROS and lipid peroxidation MDA increased in a dose-dependent manner after the cells were exposed to metformin for 48 h (Fig. 2A-C). As shown in Fig. 2D-F, with the treatment of different doses of metformin, the antioxidant GSH T-SOD and CAT levels were significantly reduced. These results implied that metformin could induce oxidative stress in lung cancer cells.

### Metformin increases the accumulation of cellular ferrous ions

**Fig. 3:**
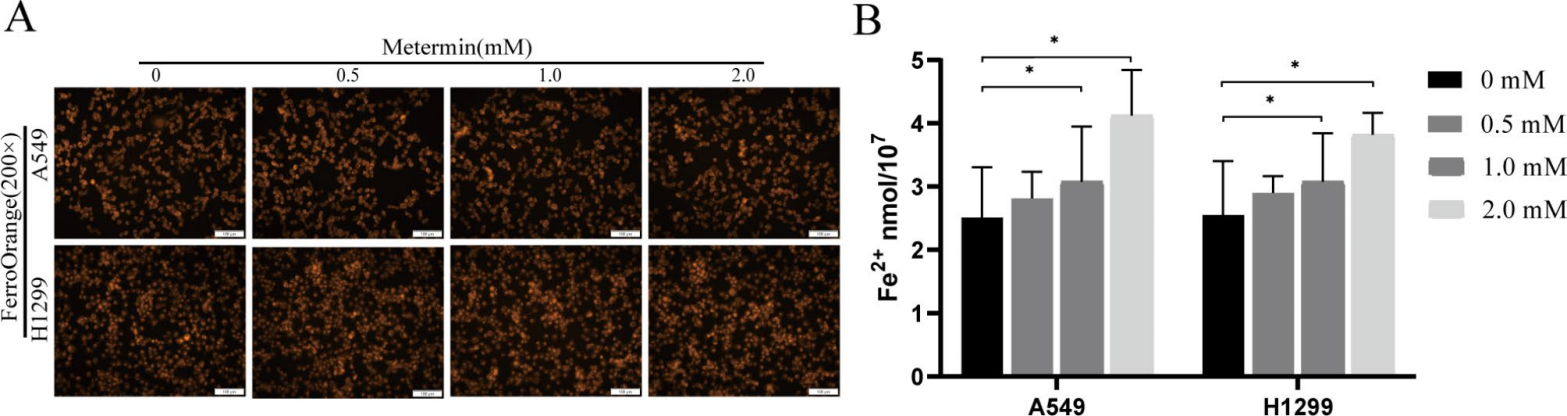
Effects of metformin on the accumulation of cellular ferrous ions. Cells were treated with different concentrations of metformin for 48 h, the intracellular ferrous ions amount were tested by a ferrous iron colorimetric assay. n = 3, ^*^P < 0.05 compared with the control group

Previous studies have reported that the changes of ROS, MDA, and GSH levels are closely associated with ferroptosis [26, 27]. To further verify that metformin treatment induced ferroptosis in lung cancer cells, the levels of ferrous iron were tested by a ferrous iron colorimetric assay kit. The results showed that the levels of ferrous iron were increased with the increase of metformin concentrations (Fig. 3, compared with the control group, the concentration of iron ion in the 2mM metformin group increased significantly; A549, *P* = 0.026; H1299, *P* = 0.035). Compared with the control group, the difference between the low concentration group and the control group was not statistically significant, which may be caused by the insufficient inducing time of metformin.

### Metformin enhanced ferroptosis by suppressing the Nrf2/HO-1 signaling pathway

**Fig. 4:**
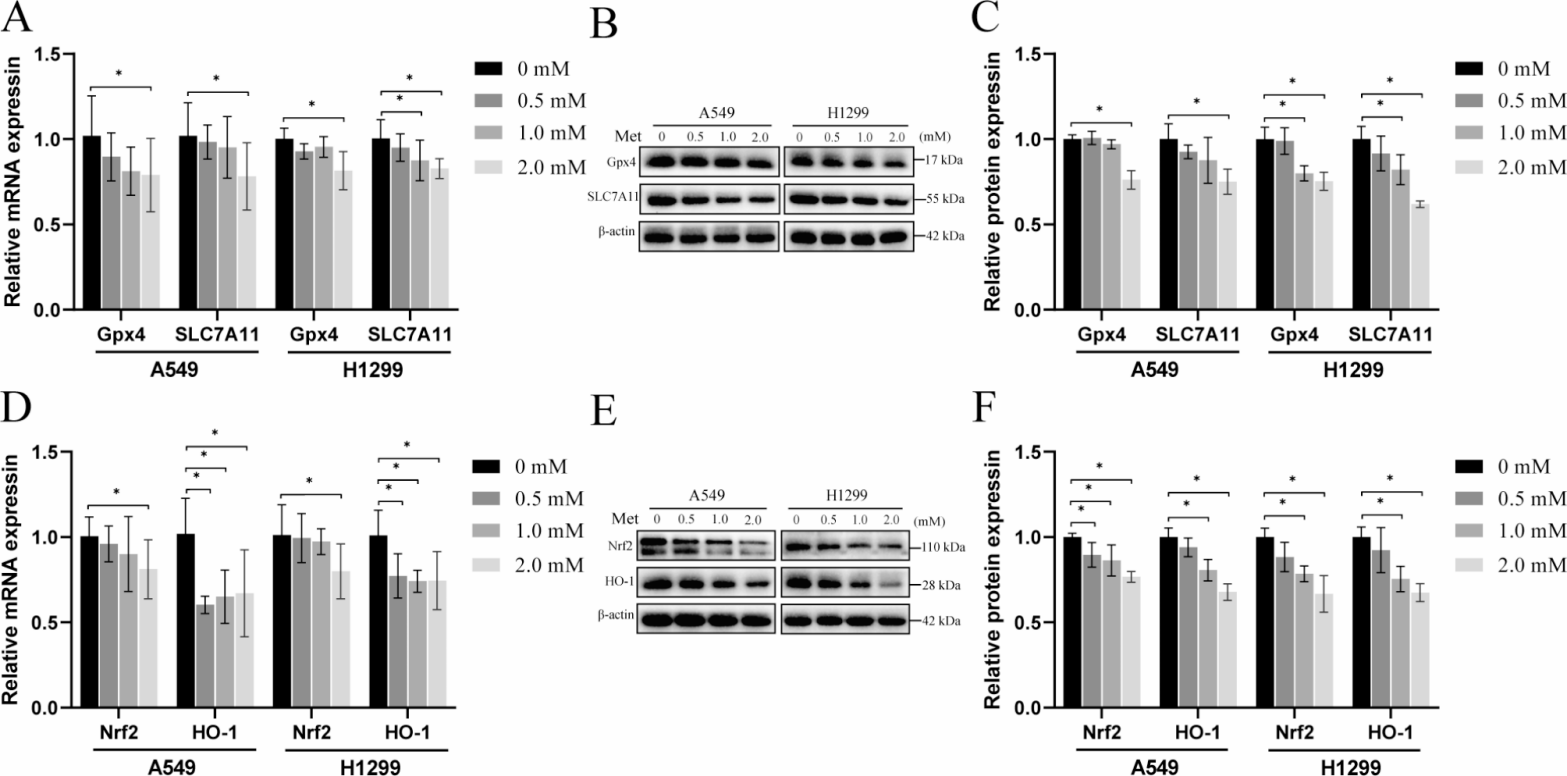
Effects of metformin on the ferroptosis-related mRNA and protein expression. **(A-C)**, the mRNA and protein expression levels of Gpx4 and SLC7A11 in A549 and H1299 lung cancer cells; **(D-F)**, the mRNA and protein expression levels of Nrf2 and HO-1 in A549 and H1299 lung cancer cells treated with different concentrations of metformin for 48 h. ^*^P < 0.05 compared with the control group

To further validate that metformin could induce ferroptosis in lung cancer cells, RT-qPCR and western blots were used to assess the expression of Gpx4, SLC7A11, Nrf2, and HO-1, proteins. As shown in Fig. 4A-C, in A549 cells, administration of 0.5mM and 1mM of metformin slightly reduced the expression of Gpx4 and SLC7A11 both in mRNA and protein levels (*P*>0.05), while administration of 2mM metformin significantly reduced the expression of Gpx4 (*P* = 0.046 for the mRNA level and P < 0.0001 for protein level, respectively) and SLC7A11 (*P* = 0.099 for the mRNA level and *P* = 0.002 for protein level, respectively). In H1299 cells, administration of 0.5mM and 1mM metformin slightly reduced the mRNA expression of Gpx4 (*P*>0.05), the 1 mM and 2 mM metformin significantly reduced the mRNA expression of SLC7A11(1 mM, *P* = 0.029; 2 mM *P* = 0.004). Notably, administration of 1mM and 2mM metformin significantly reduced the protein expression of Gpx4(1 mM, *P* = 0.001; 2 mM *P* < 0.0001) and SLC7A11(1 mM, *P* = 0.007; 2 mM *P* < 0.0001) in H1299 cells.

In Nrf2/HO-1 signaling pathway analysis (Fig. 4.D-F), RT-qPCR results showed that 2 mM of metformin significantly reduced the mRNA expression of Nrf2 in A549 cells(*P* = 0.048) and in H1299 cells(*P* = 0.019). However, 0.5 mM, 1.0 mM and 2.0 mM metformin significantly reduced the mRNA expression of HO-1 both in A549 cells and H1299 cells. Western-blot results showed 1mM and 2mM metformin significantly reduced the expression of Nrf2 and HO-1(Fig. 4.E-F). These data indicated that metformin enhanced ferroptosis involving the Nrf2/HO-1 signaling pathway in lung cancer cells.

### Fer-1 reversed metformin-induced ferroptosis in lung cancer cells

**Fig. 5:**
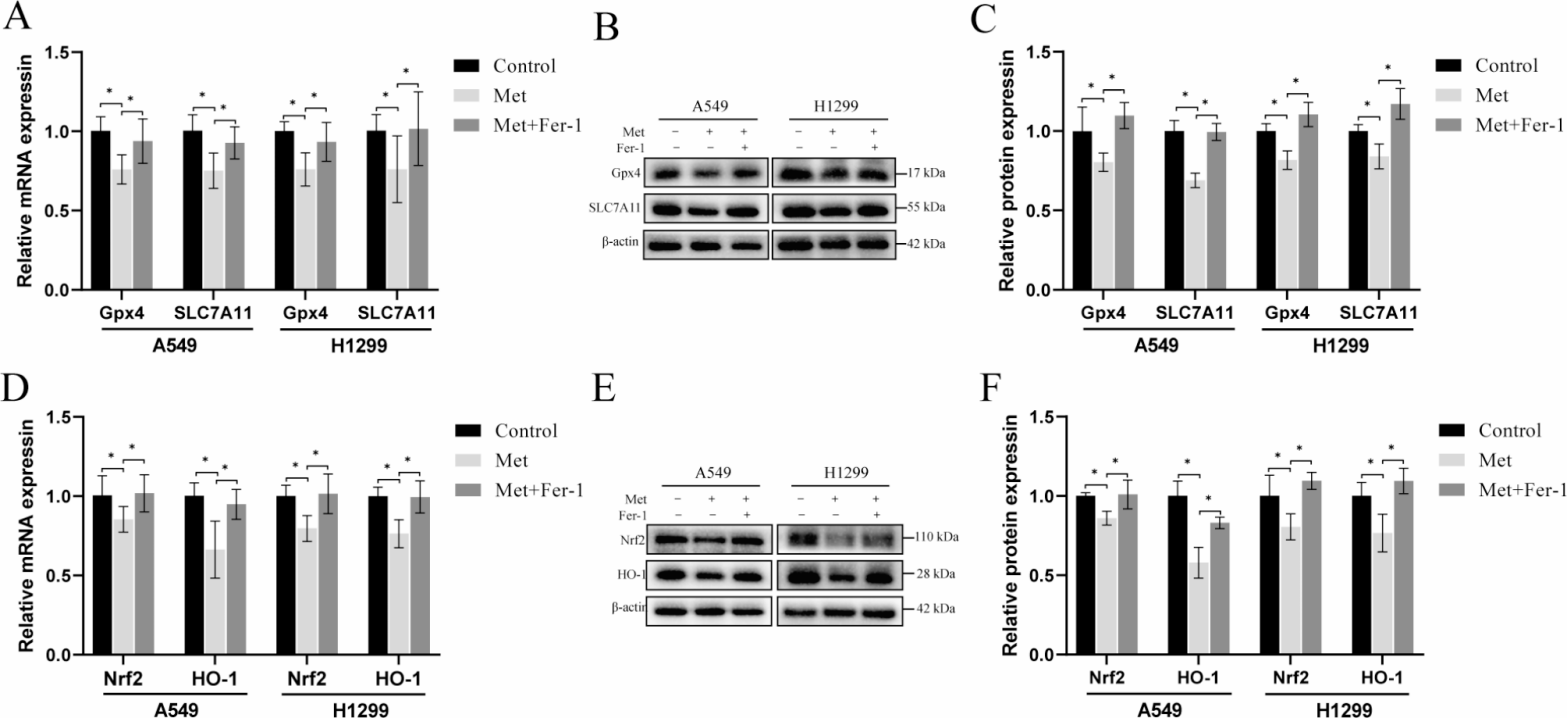
Effects of metformin and ferroptosis inhibitor ferrostatin-1 (Fer-1) on the ferroptosis-related mRNA and protein expression. **(A-C)**, the mRNA and protein expression levels of Gpx4 and SLC7A11 in A549 and H1299 lung cancer cells treated with metformin and/or Fer-1 for 48 h; **(D-F)**, the mRNA and protein expression levels of Nrf2 and HO-1 in A549 and H1299 lung cancer cells treated with metformin and/or Fer-1 for 48 h. ^*^P < 0.05 compared with the control group

A small-molecule inhibitor of ferroptosis, Fer-1, was used to confirm our metformin induced ferroptosis. As shown in Fig. 5, the decreased mRNA and protein expression Nrf2, HO-1, Gpx4, and SLC7A11 induced by 2.0 mM metformin were reversed in the presence of 10 µM Fer-1. These results indicate that Fer-1 can promote reversal of metformin-induced ferroptosis in A549 and H1299 cells.

## Discussion

Adjuvants or treatment strategies that can stimulate cell death pathways, especially non-apoptotic cell death pathways such as pyroptosis, necroptosis, and ferroptosis, may increase the anti-cancer therapeutic efficiency [13, 14]. Among them, the activation of ferroptosis may be an alternative strategy to overcome the drug resistance of traditional cancer therapies and it is reported effective in the treatment of breast cancer, pancreatic cancer, lung cancer, ovarian cancer, brain tumor, hepatocellular carcinoma, fibrosarcoma etc. [19, 28]. Studies reported that the anti-diabetic drug, metformin, could induce ferroptosis through different signaling pathways in breast cancer and hepatocellular carcinoma [21–23, 29], indicating that metformin can be used as a ferroptosis inducer for cancer treatment.

Ferroptosis is an iron-dependent nonapoptotic cell death driven by lipid peroxide [30, 31]. In this study, we determined that metformin reduced the survival of lung cancer cells (Fig. 1), induced oxidative stress through increasing intracellular ROS and MDA levels while reducing intracellular GSH, T-SOD and CAT contents (Fig. 2), and increased the accumulation of cellular ferrous ions. In addition, western blot analysis showed that the expression levels of GPX4, SLC7A11, Nrf2 and HO-1 were down-regulated by metformin treatment and the reduction levels were reversed by ferroptosis inhibitor Fer-1(Figs. 4 and 5). GPX4 and SLC7A11 are central regulators and markers of ferroptosis [32]). Nrf2, one of the most important transcription factors that regulates ferroptosis, could bind to the ARE of the SLC7A11 promoter, promoting the synthesis of GSH [33]. As a regulator of the antioxidant system, Nrf2 regulates oxidative stress via regulating the expression of HO-1. Besides, Nrf2 can effect the heme-binding ferrous content by regulating intracellular heme synthesis and metabolism [34].These results indicated that metformin induced ferroptosis of lung cancer cells through Nrf2/HO-1 signaling axis, yet the specific mechanism through which metformin regulates the Nrf2/HO-1 signaling pathway and ferroptosis in lung cancer is unclear, and whether the activation or silencing of genes of Nrf2/HO-1 signaling pathway could change the metformin-induced ferroptosis remains to be further studied.

Lung cancer is one of the main causes of cancer-related deaths worldwide. The current treatment options for lung cancer include surgery, chemotherapy, radiation therapy, targeted therapy, and immunotherapy [3]. However, due to the fact that most lung cancer patients are already in the locally advanced or advanced stage at the time of diagnosis, some patients have lost the opportunity to undergo surgery at the initial diagnosis, and many traditional radiotherapy and chemotherapy have significant side effects as a result of their lack of specificity [35, 36]. Therefore, the development of new therapeutic drugs is of great importance for increasing the patient survival. This study confirmed that metformin could induce ferroptosis in lung cancer cells by increasing oxidative stress and inhibiting the Nrf2/HO-1 signaling pathway, expanding the potential clinical application range of the cheap drug metformin, providing potential therapeutic agents for lung cancer patients.

## Conclusion

These results demonstrated that metformin induce ferroptosis through increase oxidative stress level and down-regulate the Nrf2/HO-1 signaling, expanding the potential clinical application of metformin, and providing potential therapeutic agents for lung cancer patients.

### Electronic supplementary material

Below is the link to the electronic supplementary material.

Supplementary Material 1

## Data Availability

All data generated or analyzed during this study are included in this published article.

## Abbreviations

CAT: Catalase
Fer-1: Ferrostatin-1
Gpx4: Glutathione peroxidase-4
GSH: Glutathione
HO-1: Heme oxygenase-1
MDA: Malondialdehyde
Met: Metformin
Nrf2: Nuclear factor erythroid 2-related factor 2
ROS: Reactive oxygen species
SLC7A11: Solute carrier family 7 member 11
T-SOD: Total Superoxide Dismutase
