# Supplementary material for: Metformin induces ferroptosis through the Nrf2/HO-1 signaling in lung cancer
Source: BMC Pulm Med. 2023 Sep 25;23:360. doi: 10.1186/s12890-023-02655-6 (PMC10521546; doi:10.1186/s12890-023-02655-6)
Supplement: Supplementary file 1 — Supplementary Material 1 [file 12890_2023_2655_MOESM1_ESM.pdf]

Additional file

Figure S1. Full length blot for Figure 4A, D.

(A) Expression levels of Gpx4, SLC7A11 were determined by western blot in Met-treated A549 or H1299 cells. This image is also the **Figure 4A** in the manuscript. (B) Expression levels of Nrf2 and HO-1 were determined by western blot in Met-treated A549 or H1299 cells. This image is also the **Figure 4D** in the manuscript. (C, D) The original images of Gpx4, SLC7A11 for A in Met-treated A549 cells (Figure 4A). (E) The original images of β-actin for A, B in Met-treated A549 cells (E1-Figure 4A, E3-Figure 4D). (F, G) The original images of Nrf2, and HO-1 for B in Met-treated A549 cells(Figure 4A). (H, I) The original images of Gpx4, SLC7A11 for A in Met-treated H1299 cells (Figure 4A). (J) The original images of β-actin for A, B in Met-treated H1299 cells (J1-Figure 4A, J4-Figure 4D). (K, L) The original images of Nrf2, and HO-1 for B in Met-treated H1299 cells (Figure 4D). The blots displayed in the red box represent the corresponding images in the manuscript.

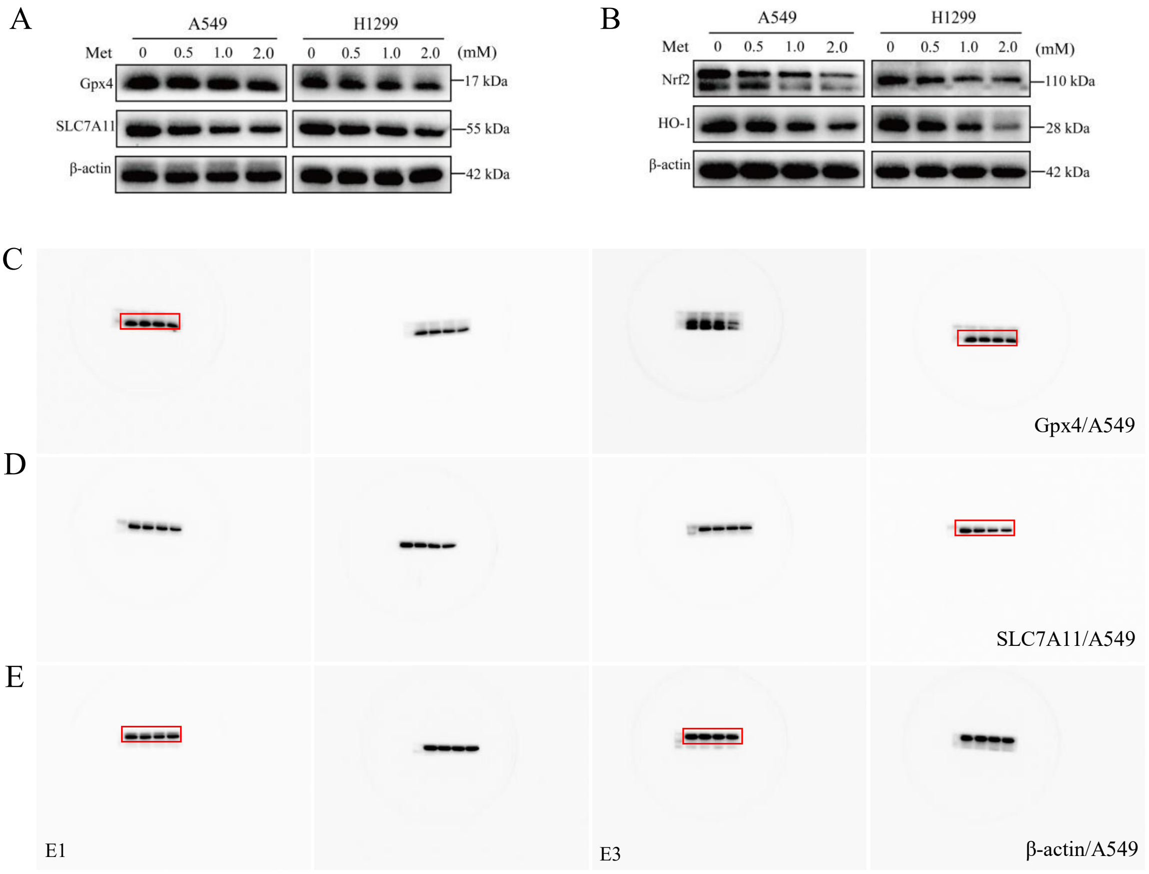

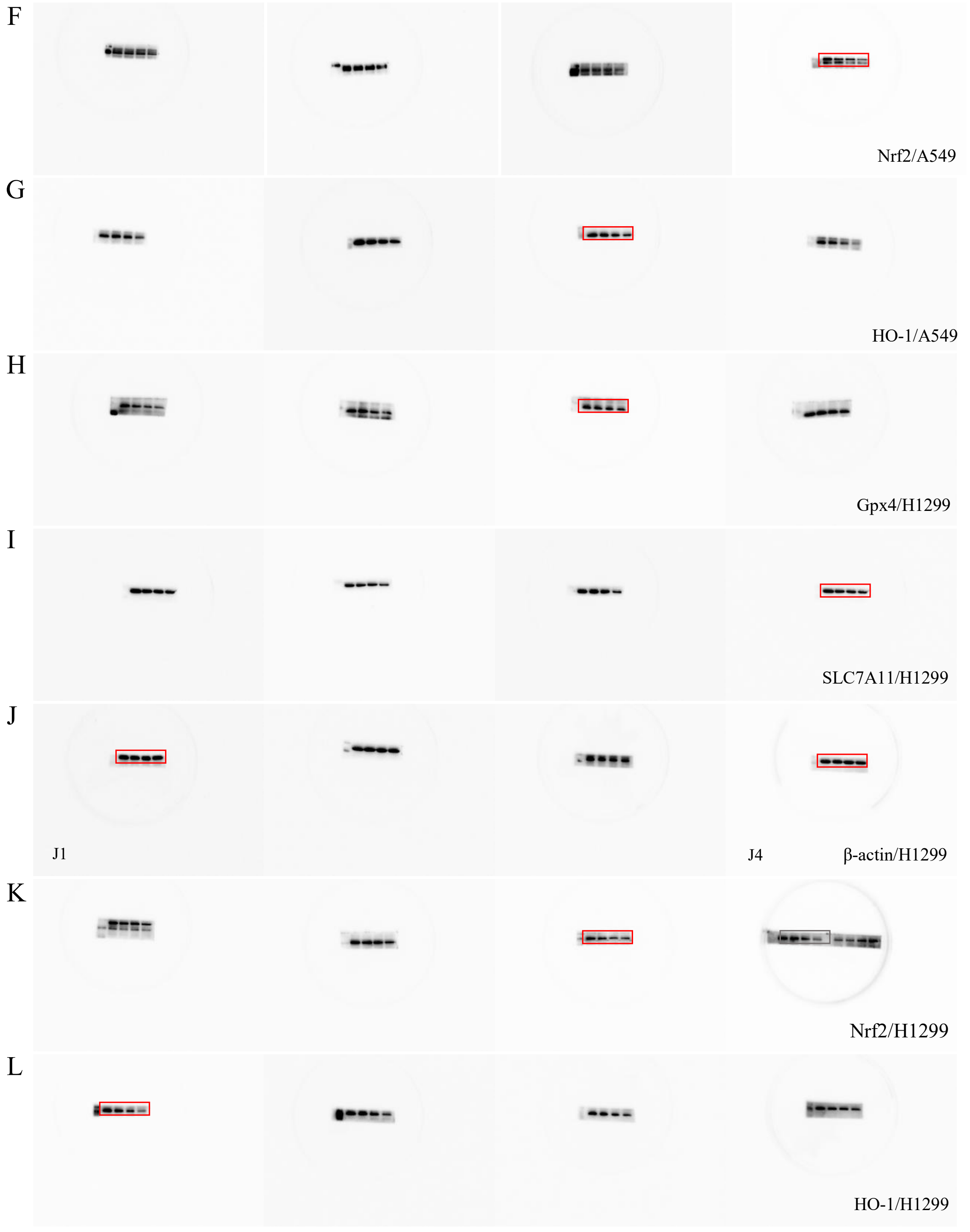
F
Nrf2/A549
G
HO-1/A549
H
Gpx4/H1299
I
SLC7A11/H1299
J
J1
J4
β-actin/H1299
K
Nrf2/H1299
L
HO-1/H1299

Figure S2. Full length blot for Figure 5A, D.

(A) Expression levels of Gpx4, SLC7A11 were determined by western blot in Met/Fer1-treated A549 or H1299 cells. This image is also the **Figure 5A** in the manuscript. (B) Expression levels of Nrf2 and HO-1 were determined by western blot in Met/Fer1-treated A549 or H1299 cells. This image is also the **Figure 5D** in the manuscript. (C, D) The original images of Gpx4, SLC7A11 for A in Met/Fer1-treated A549 cells (Figure 5A). (E) The original images of β-actin for A, B in Met/Fer1 -treated A549 cells (E2-Figure 5A, E1-Figure 5D). (F, G) The original images of Nrf2, and HO-1 for B in Met/Fer1-treated A549 cells. (Figure 5A). (H, I) The original images of Gpx4, SLC7A11 for A in Met/Fer1-treated H1299 cells (Figure 5A). (J) The original images of β-actin for A, B in Met/Fer1-treated H1299 cells (J2-Figure 4A, J3-Figure 5D). (K, L) The original images of Nrf2, and HO-1 for B in Met/Fer1-treated H1299 cells (Figure 5D). The blots displayed in the red box represent the corresponding images in the manuscript.

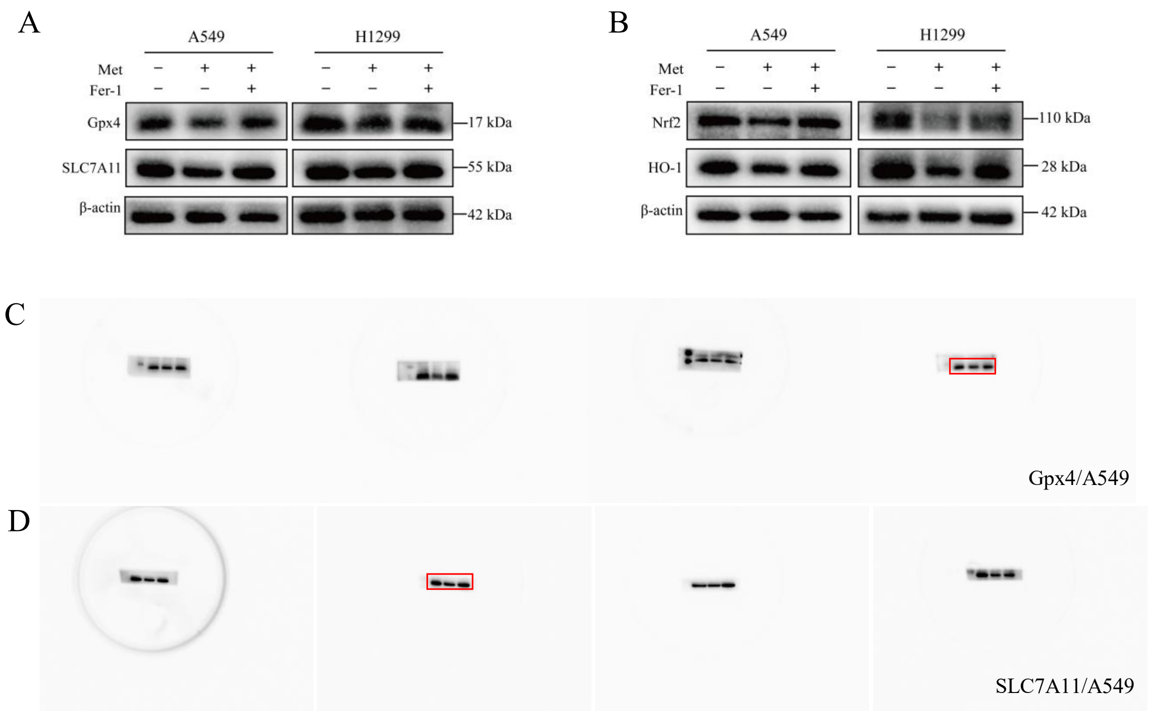

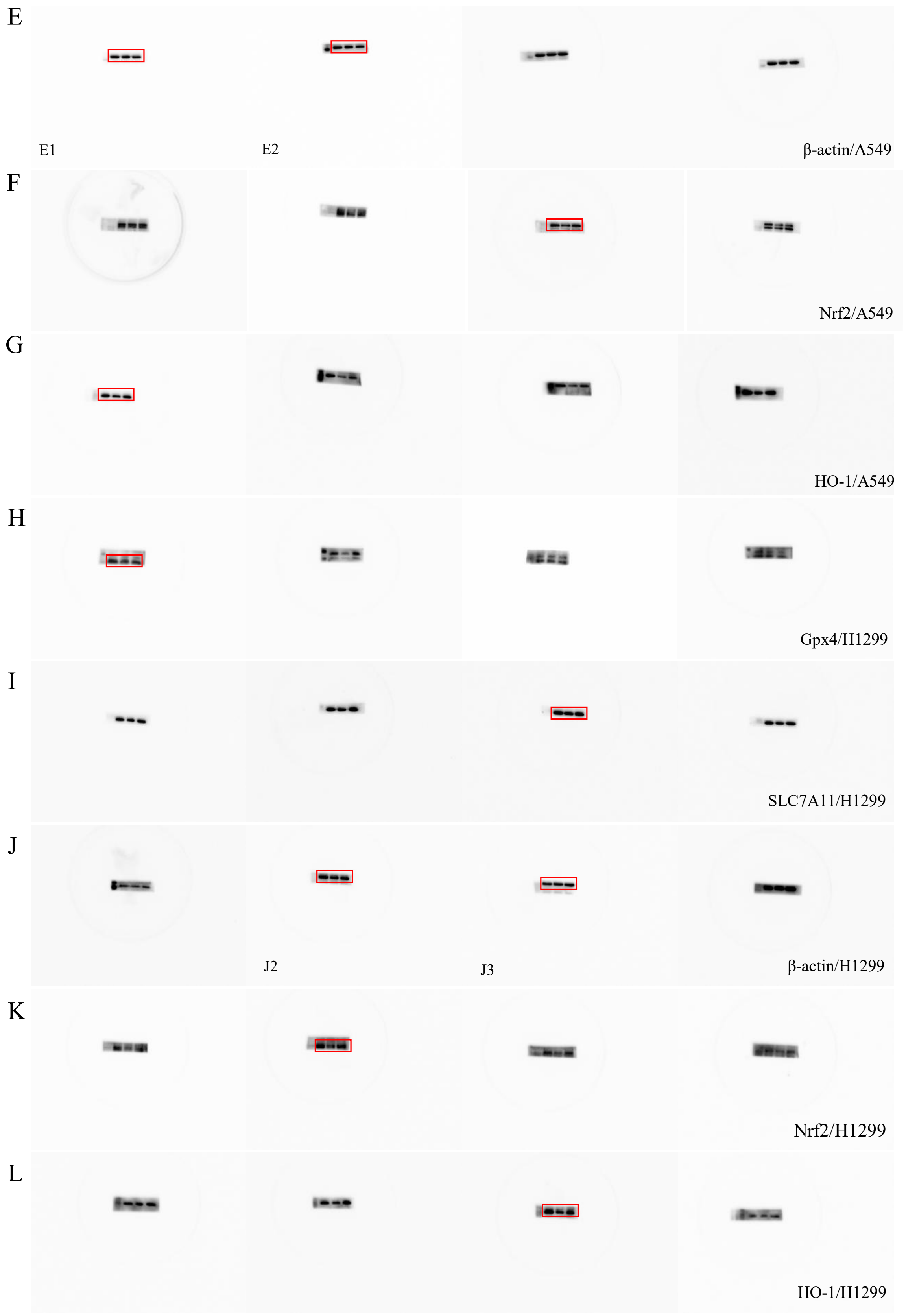
E
E1
E2
β-actin/A549
F
Nrf2/A549
G
HO-1/A549
H
Gpx4/H1299
I
SLC7A11/H1299
J
J2
J3
β-actin/H1299
K
Nrf2/H1299
L
HO-1/H1299
